# Exciton localization in solution-processed organolead trihalide perovskites

**DOI:** 10.1038/ncomms10896

**Published:** 2016-03-21

**Authors:** Haiping He, Qianqian Yu, Hui Li, Jing Li, Junjie Si, Yizheng Jin, Nana Wang, Jianpu Wang, Jingwen He, Xinke Wang, Yan Zhang, Zhizhen Ye

**Affiliations:** 1 State Key Laboratory of Silicon Materials, School of Materials Science and Engineering, Zhejiang University, Hangzhou 310027, China; 2 Center for Chemistry of High-Performance and Novel Materials and State Key Laboratory of Silicon Materials, Department of Chemistry, Zhejiang University, Hangzhou 310027, China; 3 Key Laboratory of Flexible Electronics (KLOFE) & Institute of Advanced Materials (IAM), Jiangsu National Synergetic Innovation Center for Advanced Materials (SICAM), Nanjing Tech University (NanjingTech), 30 South Puzhu Road, Nanjing 211816, China; 4 Department of Physics, Capital Normal University, Beijing Key Lab for Metamaterials and Devices, and Key Laboratory of Terahertz Optoelectronics, Ministry of Education, Beijing 100048, China

## Abstract

Organolead trihalide perovskites have attracted great attention due to the stunning advances in both photovoltaic and light-emitting devices. However, the photophysical properties, especially the recombination dynamics of photogenerated carriers, of this class of materials are controversial. Here we report that under an excitation level close to the working regime of solar cells, the recombination of photogenerated carriers in solution-processed methylammonium–lead–halide films is dominated by excitons weakly localized in band tail states. This scenario is evidenced by experiments of spectral-dependent luminescence decay, excitation density-dependent luminescence and frequency-dependent terahertz photoconductivity. The exciton localization effect is found to be general for several solution-processed hybrid perovskite films prepared by different methods. Our results provide insights into the charge transport and recombination mechanism in perovskite films and help to unravel their potential for high-performance optoelectronic devices.

## Abstract (web summary)

The recombination dynamics of photogenerated carriers in organolead trihalide perovskites are not well understood. Here, He *et al.* report that the recombination of photogenerated carriers in solution-processed methylammonium-lead-halide films is dominated by excitons weakly localized in band tail states.

## Graphical abstract

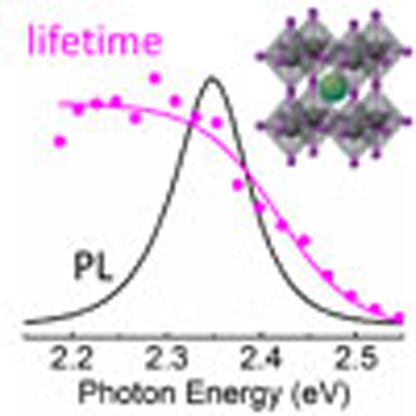

Recently, organolead trihalide perovskites have been utilized in low-temperature solution-processed photovoltaics^1^,^2^,^3^,^4^,^5^,^6^ and light-emitting devices^7^,^8^,^9^,^10^. Certified power conversion efficiency approaching 20.1% has been realized^6^. The impressive photovoltaic performance is believed to originate from the long-distance and balanced diffusion of charge carriers^11^,^12^. Remarkably, the solution-processed perovskite films also exhibit superior luminescence properties. Optically pumped lasing with low thresholds and tunable wavelengths^7^, and bright light-emitting diodes^9^,^10^ have been demonstrated.

Despite these remarkable advances, knowledge of the photophysical properties of the perovskites is still lacking. One of the key questions concerns the recombination dynamics of photogenerated charges: whether exciton or free carrier (FC) is the dominant recombination channel in organolead trihalide perovskites. The answer will help to interpret the seemingly counterintuitive facts that organolead trihalide perovskites can act both as extraordinary photovoltaic materials and superior gain mediums for lasing^13^. In general, photovoltaic materials require efficient separation of photocarriers, and lasing materials require high recombination rates. The reported exciton binding energy of the perovskites^14^,^15^ is comparable to the thermal energy at room temperature (RT), which arouses arguments that in such a case excited states will tend to dissociate into FCs rather than recombine radiatively.

Several groups have used photoluminescence (PL) to study the competition between exciton and FC in organolead trihalide perovskites. In a few steady-state PL studies, the RT PL was attributed to exciton recombination,^16^,^17^,^18^ but the conclusions lacked solid evidence. Recently, several groups^8^,^14^,^19^,^20^ attributed the RT PL to FC recombination. For example, D'Innocenzo *et al.*^14^ argued that excitons generated by low-density excitation are almost fully ionized at RT when the exciton binding energy is moderately larger than the RT thermal energy. The band filling effect^8^ and quadratic dependence of the PL intensity on the excitation intensity^19^, the two characteristic features of FC recombination, were observed at relatively high excitation levels. We note that the observation of FC recombination at relatively high excitation levels is not surprising because the reduced exciton binding energy originated from the screening effect of FCs, a phenomenon that has been well established in many semiconductors^21^,^22^.

Here we show that under an excitation level close to the working regime of solar cells, the radiative recombination of photogenerated carriers in solution-processed CH_3_NH_3_PbX_3_ perovskites is dominated by excitons localized in band tail states. The excitonic nature of the emission is evidenced by the excellent power-law dependence of the PL intensity on the excitation intensity expected for bound excitons, and is supported by the PL lineshape analysis. The localization effect is supported by the spectral dependence of the PL lifetime and frequency-dependent THz photoconductivity results. We also show that the exciton localization effect is general in several solution-process perovskite films.

## Results

### Evidence for exciton localization in CH_3_NH_3_PbBr_3_ films

We use solution-processed CH_3_NH_3_PbBr_3_ films for the PL studies. The films show good crystalline and optical quality (Supplementary Fig. 1). To avoid degradation induced by air exposure, all samples were prepared in a nitrogen-filled glove box, coated with a polymethyl methacrylate (PMMA) layer and were measured in vacuum (10^−1^ Pa). The CH_3_NH_3_PbBr_3_ film shows emission at 2.35 eV, in agreement with the reported values^23^,^24^.

**Figure 1:**
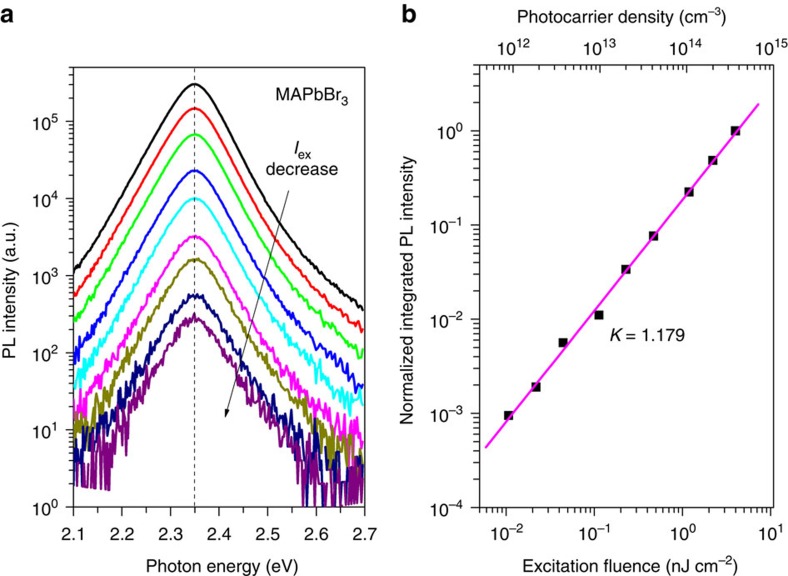
Excitation density-dependent PL of solution-processed CH_3_NH_3_PbBr_3_ films. (**a**) Steady-state PL spectra recorded with excitation density from 0.01 to 4 nJ cm^−2^. All spectra are measured in vacuum at RT. In all spectra, the peak energy (indicated by the dashed line), lineshape and linewidth are identical within the experimental error. (**b**) Logarithm plot of the integrated PL intensity versus excitation density. The data show a power-law dependence with *k*=1.179.

Near-band-edge emission in semiconductors may have several origins, including exciton recombination, FC recombination (also known as band-to-band transition), free-to-bound recombination and donor–acceptor pair recombination. To determine which process is dominant in our samples, we measured the PL spectra under various excitation densities close to or lower than the photovoltaic working regime (∼5 × 10^14^ cm^−3^) (refs 14, 25). The PL lineshapes in Fig. 1a are almost identical in the whole range of excitation intensity. The PL intensity shows excellent power-law dependence on the excitation power, with a power-law exponent of 1.179. In direct bandgap semiconductors and under non-resonant excitation conditions, the integrated PL intensity (*I*_PL_) is a power-law function of the excitation density^26^,


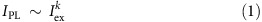


with *k*=2 for FC recombination, 1<*k*<2 for recombination of excitons (including free excitons and bound excitons) and *k*<1 for free-to-bound recombination and donor–acceptor pair. The model was further refined by Shibata *et al*^27^, who provided an analytical formula to confirm that 1<*k*<2, even for free excitons. The physics behind the process is the photo-neutralization of the donors/acceptors, which are present in all semiconductors and result in competitive recombination channels. Our *k* value agrees well with those reported for excitons in semiconductors^26^,^27^.

**Figure 2:**
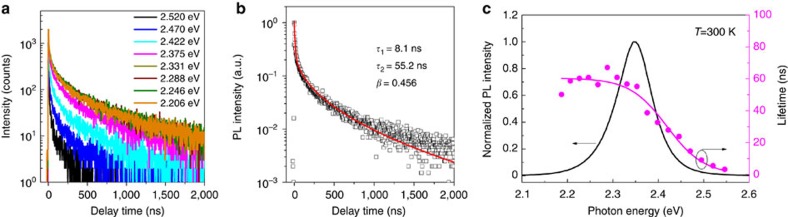
Spectral-dependent PL decay of solution-processed CH_3_NH_3_PbBr_3_ films. (**a**) PL decay curves monitored at various emission energies. The lifetime decreases markedly on the high-energy side of the emission. (**b**) Typical fitting of a decay curve by the thermalized stretching exponential model described by equation (2). (**c**) The lifetime of localized excitons *τ*_2_ (circles) as a function of emission energy. The data are fitted with equation (3) (magenta line), with the lifetime of localized exciton *τ*_LE_=60.5 ns, mobility edge of 2.419 eV and the localization energy *E*_0_=40.9 meV.

Figure 2a shows the RT PL decay curves monitored at different excitation energies. On the low-energy side, the PL lifetime is almost constant for all emission energies. On the high-energy side, the PL lifetime decreases with increasing emission energy. The PL decay curves can be well fitted by the thermalized stretching exponential line shape^28^,^29^


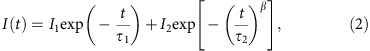


where *τ*_*i*_ is the decay time and *I*_*i*_ is the weight factor of each decay channel. A typical fitting result is plotted in Fig. 2b (for all fitting results, see Supplementary Fig. 2). The fitting curves do not match normal mono-exponential or simple stretched-exponential decay (Supplementary Fig. 2c). Stretched-exponential decay is regarded as evidence of the exciton localization, in which the parameter *β* is related to the dimensionality of the localizing centres. The former exponential term in equation (2) represents the relaxation of free or extended states towards localized states, whereas the latter stretched-exponential term accounts for the communication between the localized states. We found that all the decay curves can be well fitted with a constant *β* of 0.43±0.03. Both lifetimes *τ*_2_ and *τ*_1_ show clear spectral dependence (Fig. 2c; Supplementary Fig. 2d). It markedly decreases on the high-energy side, while remaining constant on the low-energy side.

The spectral dependence of *τ*_2_ can be described by a well-established model^30^ for excitons localized in the tail states


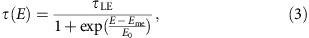


where *τ*_LE_ is the lifetime of localized excitons, *E*_me_ can be regarded as the mobility edge and *E*_0_ is a characteristic energy of the density of band tail states, which can be a measure of the localization energy. The best fit of the data gives *τ*_LE_=61 ns, *E*_me_=2.419 eV and *E*_0_=41 meV. The localization energy, 41 meV, is higher than the RT thermal energy, which is consistent with localized excitons being observed even at RT.

We provide evidence to show that the PL in our perovskite films is not due to FC recombination but to a localization effect. The first evidence comes from the PL spectra under high excitation. As shown in Supplementary Fig. 3, increasing the excitation density leads to the occurrence and increase of a higher energy tail at ∼2.41 eV. The result suggests that the main peak at 2.32 eV does not originate from FC recombination because FC emission has the highest energy among all radiative recombination. With increasing excitation density, the 2.32 eV peak is still dominant over the 2.41 eV FC emission and is almost unshifted. The unshifted luminescence agrees with the reported^31^ feature of localized excitons, which means that the many-particle effect of these localized excitons is weak. The second evidence comes from the frequency-dependent THz measurements (the experimental details can be found in Supplementary methods). Supplementary Figure 4 plots the photoconductivity induced by 400-nm pump pulses. The real part of the induced photoconductivity Δ*σ*_1_(*ω*) decreases with decreasing frequency, and the imaginary part Δ*σ*_2_(*ω*) has a negative value at low frequency. The results do not support the Drude model for FCs^32^, which predicts increasing Δ*σ*_1_(*ω*) with decreasing frequency and positive Δ*σ*_2_(*ω*). The real part increasing with frequency and the negative imaginary part are typical signatures of carrier localization^33^,^34^. The low conductivity at low energy is consistent with the insulating nature of the charge-neutral excitons^32^. Such features build up immediately after excitation (Δ*t*=3 ps) and are maintained within the entire timescale (Δ*t*=300 ps).

**Figure 3:**
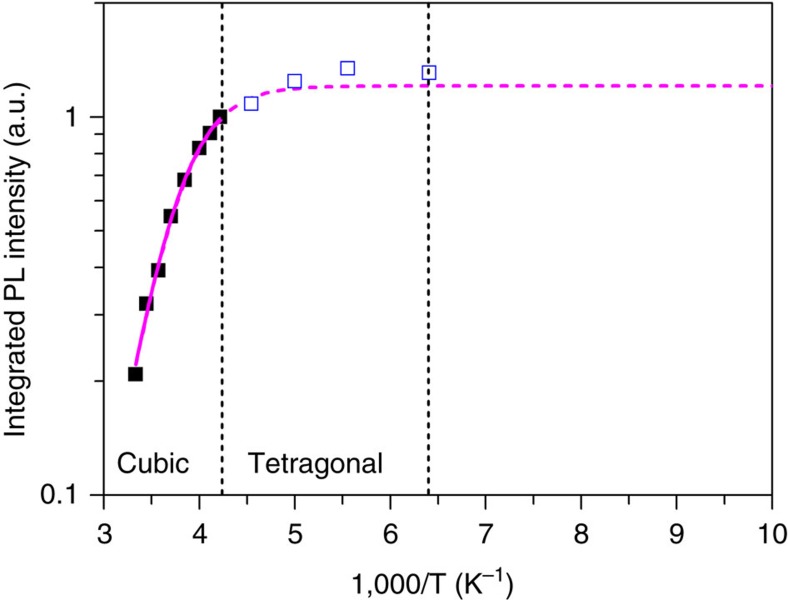
PL thermal quenching behaviour of solution-processed CH_3_NH_3_PbBr_3_ film. Integrated PL intensity as a function of the reciprocal temperature under low excitation (photocarrier density ∼3.7 × 10^14^ cm^−3^). There is a cubic (RT) to tetragonal phase transition at 236 K, and tetragonal-to-tetragonal phase transition at 155 K. The magenta solid curve represents the fitting result for the cubic data (Supplementary Note 1). The magenta dashed curve is the extrapolation result. The tetragonal data (open square) are plotted for comparison.

The above results indicate that the PL decay is dominated by recombination of localized excitons rather than FCs at RT. We also conducted excitation density-dependent PL and spectral-dependent lifetime measurements on CH_3_NH_3_PbBr_3_ films at 237 K. These experiments were designed to check whether the assignment of localized excitons is valid for the perovskite films at low temperatures, which have higher quantum efficiency than that of the films at RT. The temperature of 237 K was chosen based on the cubic-to-tetragonal phase transition^35^ of CH_3_NH_3_PbBr_3_. As shown in Fig. 3, the relative internal quantum efficiency (IQE) at 237 K is estimated to be ∼80% based on the temperature-dependent PL intensity (Supplementary Note 1), a method that has been widely used in inorganic compound semiconductors^36^. The results (Supplementary Fig. 5) show that the *k* value and PL decay features at 237 K are similar to those at RT, indicating that localized excitons still dominate the PL under the condition of high quantum yield.

Recently, there have been arguments that at RT excitons cannot exist in perovskites because the dielectric constant of perovskite materials is very large^37^,^38^,^39^,^40^, which results in dielectric screening and consequent dissociation of excitons. However, our evidence for exciton localization suggests that the role of the dielectric screening effect might be more complicated than expected. In fact, excitons do exist in materials with large dielectric constants. In ferroelectric oxides, such as SrTiO_3_ and FeBiO_3_, it is accepted that self-trapped excitons^41^ and charge transfer vibronic excitons may exist^42^. The mechanism for these excitons can be quite different from the normal one. For example, charge transfer vibronic excitons is correlated electronic and hole polarons induced by the Jahn–Teller type lattice distortion, which are localized and can be quite stable at RT^42^. In view of these facts, we suggest that excitons can also exist in trihalide perovskites, despite the possible large dielectric constant under illumination. As additional experimental evidence, the absorption of exciton resonance is observed in the optical absorption spectra of CH_3_NH_3_PbBr_3_ film (Supplementary Fig. 1b). Recent analysis of the absorption spectra suggested that bound exciton states also exist in CH_3_NH_3_PbI_3_ films^15^.

The properties of perovskite films may depend on the preparation methods and/or processing conditions. To test whether the exciton localization is a general mechanism, we prepared CH_3_NH_3_PbBr_3_ films by the two-step method and measured the excitation-dependent PL and spectral-dependent lifetime (Supplementary Fig. 6). The samples also exhibit features similar to that shown in Fig. 2 and Fig. 3, indicating the localized exciton nature.

### Exciton localization in CH_3_NH_3_PbI(Cl)_3_ films

**Figure 4:**
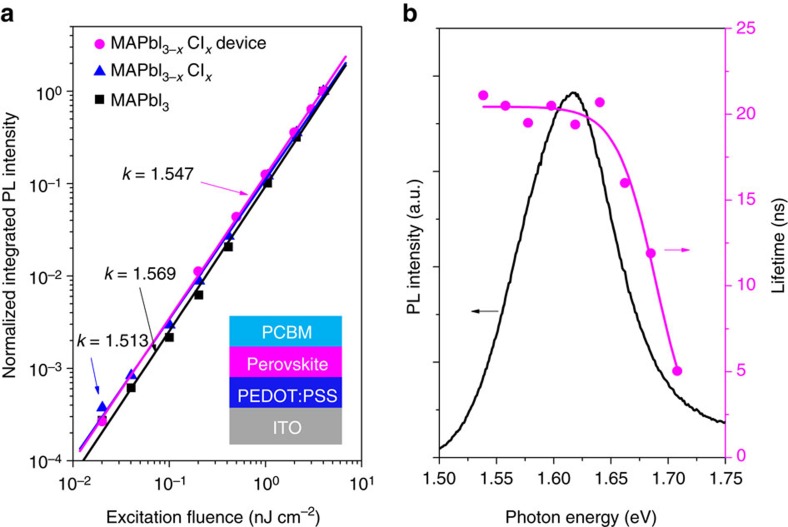
Steady-state PL spectra and transient PL decay of CH_3_NH_3_PbI_3_ and CH_3_NH_3_PbI_3−*x*_Cl_*x*_. (**a**) Logarithm plot of the integrated PL intensity versus excitation density for CH_3_NH_3_PbI_3_ and CH_3_NH_3_PbI_3−*x*_Cl_*x*_ films. Insert is a typical photovoltaic structure with a CH_3_NH_3_PbI_3−x_Cl_x_ film sandwiched between two charge-transporting interlayers. The data show good power-law dependence with *k* values of 1.569, 1.513 and 1.547. (**b**) PL lifetime (solid circles) of ITO/PEDOT:PSS/ CH_3_NH_3_PbI_3−*x*_Cl_*x*_/PCBM photovoltaic structure as a function of the emission energy. The data are fitted with equation (3) (magenta line), with the lifetime of localized exciton *τ*_LE_=20.5 ns, mobility edge 1.689 eV and localization energy *E*_0_=17.3 meV. The PL lifetime is greatly reduced due to the quenching effects of the adjacent layers.

The emissions from solution-processed CH_3_NH_3_PbI_3_ and CH_3_NH_3_PbI_3−*x*_Cl_*x*_ films are also dominated by localized excitons. The power-law dependence of the PL intensity on the excitation density reveals *k* values of ∼1.5 (Fig. 4a), and the PL decay shows spectral dependence (Supplementary Fig. 7) similar to that observed in CH_3_NH_3_PbBr_3_. The *k* values are larger than the value obtained in CH_3_NH_3_PbBr_3_. According to the theory developed by Schmidt *et al.*^26^ and Shibata *et al.*^27^, such a difference mainly represents the different material properties such as the probabilities of radiative recombination and competitive nonradiative recombination. For example, the decrease of crystal perfection is expected to increase the *k* value. Moreover, the contribution of FC recombination may also lead to a change of *k* value. The PL lineshape analysis (Supplementary Fig. 8; Supplementary Note 2) indicates a small fraction (∼9.5%) of FC recombination in the emission of CH_3_NH_3_PbI_3−*x*_Cl_*x*_ film, which is reasonable because the reported exciton binding energy^43^ of CH_3_NH_3_PbI_3_ and CH_3_NH_3_PbI_3−*x*_Cl_*x*_ is lower than CH_3_NH_3_PbBr_3_, so the thermal dissociation of excitons is easier. The coexistence of exciton and FC recombination in the PL spectra has been observed in other materials^44^ with exciton binding energy comparable to the RT thermal energy. Excitation-dependent PL at low temperature reveals smaller *k* values (Supplementary Fig. 9), which can be interpreted by reduced thermal dissociation of excitons at low temperature.

It is of interest to determine whether the conclusion of localized excitons in perovskite materials is valid for the same material in a photovoltaic device structure. We construct such a structure using CH_3_NH_3_PbI_3−*x*_Cl_*x*_, as shown in the inset of Fig. 4a. The PL intensity still shows power-law dependence on the excitation density, with a *k* value of 1.547, very close to the result of the bare film. Moreover, the PL decay spectra (Supplementary Fig. 10) show dependence on the emission energy and the lifetime can be well described by equation (3) with *E*_0_ ∼17 meV, as seen in Fig. 4b. The results indicate that the PL of CH_3_NH_3_PbI_3−*x*_Cl_*x*_ film in a typical photovoltaic structure is also dominated by the localized excitons rather than FC recombination.

## Discussion

**Figure 5:**
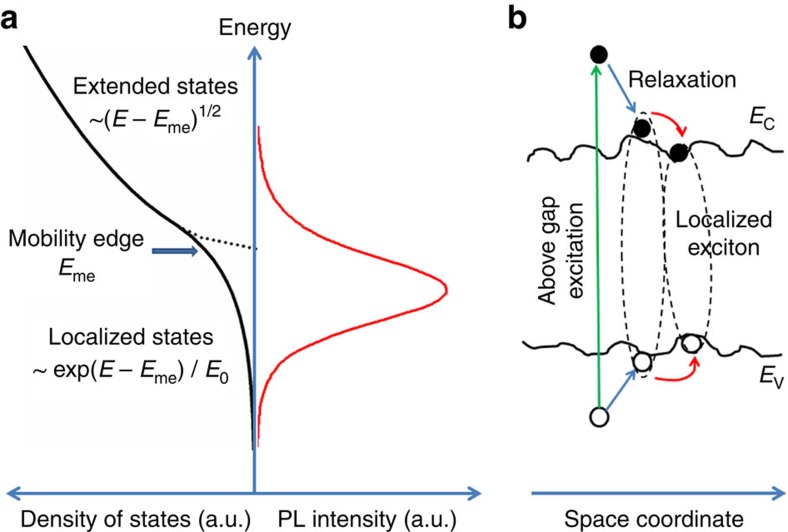
Physical picture of exciton localization. (**a**) The density of the extended and localized states, and emission in perovskites (schematic). The density of the localized states is approximated by an exponential tail with the form of ∼exp(−*E*/*E*_0_). The localized and extended states are divided by the mobility edge. Under low excitation, the excitons mainly occupy the localized states. Under high excitation, the localized states can be filled and the photocarriers also occupy the extended states, leading to emissions of free carriers. (**b**) Schematic drawing of exciton localization in space coordinates. With the presence of structural disorder, the tails of the localized states form local potential fluctuation in the energy bands. These potential minima can localize electrons and holes to form localized excitons. The carriers can transfer between the local potential minima, leading to long PL lifetime.

The physical picture of the recombination of localized excitons is illustrated in Fig. 5. The density of localized states is approximated by an exponential tail with the form of ∼exp(−*E*/*E*_0_). The excitons can be either partly localized (one carrier is localized with another carrier bound to it by Coulomb attraction) or wholly localized^21^. With increasing energy, the localized excitons may transit to the extended exciton states (approaching free excitons) at the transition region known as the mobility edge. Under low excitation, most of the photocarriers occupy the tail states. The picture can also be understood in the space coordinates as shown in Fig. 5b. In this case, the tail states are represented by the local potential minima in the conduction and/or valence bands. The photogenerated carriers transfer to these potential minima to form localized excitons, which have much longer lifetime than free excitons due to the transfer between localized states^28^,^29^. The long lifetime of the localized excitons accounts for the observed long PL lifetime. This phenomenon is also observed in inorganic semiconductors. For example, localized exciton lifetime as long as 65 ns at RT has been reported^29^ in InGaN.

Tail states are very common in semiconductors and can be induced by doping, compositional changes and structural deformation^21^,^45^. Although solution-processed perovskites are materials with reasonable crystal quality, structure imperfections are inevitable. For example, the large rotational freedom of the polar CH_3_NH_3_^+^ cation can produce structural disorder^37^,^46^. Unintentional/intentional doping is possible. The weak bonding between lead and halogens may also produce local disorder, especially in the surface and crystal boundary region. Recent studies^47^ revealed that the grain boundaries exhibited faster nonradiative decay. Other results^48^ suggest that perovskites with larger grains exhibit better photovoltaic performance. Given these facts, it is reasonable to suggest the existence of tail states in solution-processed perovskite films.

The energy and intensity of localized state-related emission depend strongly on the nature (for example, energy level) and density of the localized states. In the case of strong localization, such as deep-level centres, the emission energy may be substantially reduced. The localized states may emit weakly provided their density is low. In the present case, strong near-band-edge emission can be realized, indicating that excitons are weakly localized in the band tail states. The weak localization is evidenced by the relatively small localization energy reflected by the *E*_0_ values in equation (3) and by the small Stokes shift of the luminescence (Supplementary Fig. 1b). The localization effect can even be beneficial to light-emitting devices. Localized exciton may contribute to the optical gain because the localized states can be easily filled, provided their density is not too high^31^,^49^,^50^. We performed temperature-dependent PL measurements under moderate excitation to check whether nonradiative channels become dominant when the excitation density increases. Supplementary Fig. 11 shows that the relative IQE of moderate excitation is much higher than that of low excitation, in agreement with the result reported by Deschler *et al*^8^. This result implies that other decay channels such as Auger recombination do not become dominant, which will be of great benefit to low threshold lasing. We conducted PL experiments at high excitation (photocarrier concentration up to ∼10^19^ cm^−3^). Amplified spontaneous emission was observed on the low-energy side (at 2.26 eV) of the localized exciton emission with a threshold of ∼300 μJ cm^−2^ (Supplementary Fig. 3b). It is noteworthy that FC emission (∼2.41 eV) emerges with increasing excitation density. These results suggest that the amplified spontaneous emission is likely from localized excitons, which indicates that the optical gain can come from the filled localized states, and it is possible to achieve a low threshold by controlling the density of localized states. Moreover, exciton localization may also increase the luminescence efficiency because the oscillator strength of the transitions in the localized states is greatly enhanced^49^,^51^.

We emphasize that the generation of localized excitons at excitation levels close to the working regime of solar cells does not conflict the fact that the hybrid perovskites are superior photovoltaic materials. The localized excitons can diffuse by a thermally activated multiple trapping–escaping process^52^,^53^. This process leads to an exponential dependence of the diffusion coefficient on the exciton energy (*E*_exc_), as well as on the temperature^53^


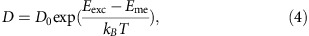


where *D*_0_ is the diffusion coefficient of the extended excitons. Taking the energetic distance *E*_exc_*−E*_me_ ∼70 meV (as extracted from Figs 2c,4b), we estimate the RT diffusion coefficient of localized excitons *D* as 0.067*D*_0_, only approximately one order of magnitude lower than the free exciton. If we invoke the reported^11^,^12^, *D* value of 0.011−0.054 cm^2^ s^−1^ for the perovskites, a *D*_0_ of ∼0.16−0.80 cm^2^ s^−1^ is obtained. This value is comparable to the reported values^53^,^54^ for CdS_1−*x*_Se_*x*_ (0.3 cm^2^ s^−1^) and In_1−*x*_Ga_*x*_N (0.5–1.1 cm^2^ s^−1^), two alloy semiconductors known as the typical examples for the study of localized excitons. In combination with the very long lifetime, a long carrier diffusion length in perovskites is expected in the scenario of exciton localization.

In summary, we have presented solid evidence that the RT PL in organolead trihalide perovskites is dominated by weakly localized excitons. The evidence includes: (i) the excellent power-law dependence of the PL intensity on the excitation intensity with 1<*k*<2, (ii) the localization effect indicated by the spectral dependence of the PL lifetime and frequency-dependent THz photoconductivity and (iii) the coexistence of exciton and FC recombination under high excitation. Exciton localization is suggested as the origin of the long PL lifetime in this class of materials. We find that the localization effect is general in solution-process perovskite films due to the presence of crystal imperfections. The localization of excitons strongly influences the transport and recombination properties of perovskite materials. The dominance of the localized exciton in the recombination channels as well as its higher IQE under moderate excitation, strongly suggests that it is possible to utilize these benefits to realize low threshold lasing in perovskites, as has been demonstrated in III–V and II–VI semiconductors and devices. The elaborate tailoring of the localization effect in perovskites is thus highly attractive in designing future high-performance optoelectronic devices.

## Methods

### Synthesis of perovskite films

All the indium-tin oxide (ITO)-coated glass substrates were cleaned sequentially in deionized water, ethanol, acetone and oxygen plasma before spin-coating. The perovskite CH_3_NH_3_PbI_3−*x*_Cl_*x*_ was prepared according to the reported procedure^11^. Methylamine iodide was prepared by reacting 33 wt % methylamine in ethanol (Sigma-Aldrich), with 57 wt % hydroiodic acid in water (Sigma-Aldrich), at RT. Hydroiodic acid was added dropwise while stirring. After drying at 100 °C, the resultant white powder was dried overnight in a vacuum oven and was recrystallized from ethanol before use. To form the CH_3_NH_3_PbI_3−*x*_Cl_*x*_ precursor solution, methylammonium iodide and lead (II) chloride (Sigma-Aldrich) were dissolved in anhydrous *N*,*N*-dimethylformamide (DMF) in a 3:1 molar ratio of methylamine iodide to PbCl_2_, with final concentrations 0.88 M lead chloride and 2.64 M methylammonium iodide. The precursor was filtered through a 220-nm polytetrafluoroethylene (PTFE) filter head, then spin-coated at 3,000 r.p.m. for 30 s on ITO-coated glass; finally, it was annealed at 95 °C for ∼10 min. CH_3_NH_3_PbI_3_ was prepared via the sequential deposition route^3^. A PbI_2_ (Sigma-Aldrich) solution in DMF (462 mg ml^−1^) was spin-coated on glass substrate and then kept at 70 °C. After drying, the films were dipped in a solution of CH_3_NH_3_I in 2-propanol (10 mg ml^−1^) for ∼60 s and rinsed with 2-propanol, and then spin-coated to form uniform CH_3_NH_3_PbI_3_ thin films. For CH_3_NH_3_PbBr_3_ preparation,^24^ CH_3_NH_3_Br was first prepared by mixing methylamine with hydrobromic acid (48% in water; CAUTION: exothermic reaction) in 1:1 molar ratio in a 100-ml flask under continuous stirring at 0 °C for 2 h. CH_3_NH_3_Br was then crystallized by removing the solvent in an evaporator, washing three times in diethyl ether for 30 min and filtering the precipitate. The material, in the form of white crystals, was then dried in vacuum at 60 °C for 24 h and was then kept in a dark, dry environment until further use. A 20-wt % solution of CH_3_NH_3_PbBr_3_ was prepared by mixing PbBr_2_ and CH_3_NH_3_Br in a 1:3 molar ratio in DMF. The precursor was spin-coated at 4,000 r.p.m. for 30 s on ITO-coated glass and was annealed at 60 °C for ∼10 min.

### Fabrication of the photovoltaic device structure

The structure of ITO/PEDOT:PSS/perovskite/PCBM was fabricated on patterned ITO-coated glass substrates (sheet resistance: 15 Ω sq^−1^). The substrates were cleaned sequentially in acetone, ethanol, deionized water and ethanol for 10 min each, followed by oxygen plasma treatment for 15 min. A poly(3,4-ethylenedioxythiophene):poly(styrenesulfonate) (PEDOT:PSS) layer was spin-coated onto the substrates at 4,000 r.p.m. for 60 s and then annealed in air at 140 °C. The sample was transferred into a glove box. Next, the CH_3_NH_3_PbI_3−*x*_Cl_*x*_ precursor solution was spin-coated at 3,000 r.p.m. for 45 s, followed by annealing on a hot plate at 100 °C for ∼60 min. The [6,6]-phenyl-C61-butyric acid methyl ester (PCBM) layers were deposited from a 30 mg ml^−1^ chlorobenzene solution at 2,000 r.p.m. for 45 s. To avoid the degradation induced by air exposure, the devices were packaged with glass.

### PL measurements

The steady-state and time-resolved PL measurements were performed on a FLS920 fluorescence spectrophotometer (Edinburgh Instruments). To minimize the effect of air exposure, all the measurements were taken in vacuum with pressure of <0.01 torr. Pulsed laser diodes (EPL405 and EPL635) with tunable repeating frequency of 20 kHz to 20 MHz were used as the excitation source. For CH_3_NH_3_PbI_3_ and CH_3_NH_3_PbI_3−*x*_Cl_*x*_ films, EPL635 with a wavelength of 638.8 nm and pulse width of 86.4 ps was used. For CH_3_NH_3_PbBr_3_, we used EPL405 with a wavelength of 404.2 nm and pulse width of 58.6 ps. The excitation fluence for both wavelengths was ∼4 nJ cm^−2^. For excitation density-dependent measurements at the low level, the lasers operated at 20 MHz and the light fluence was tuned by a neutral attenuator. For moderate excitation experiments, a 355 nm frequency-tripled Nd:YAG laser (FTSS 355-50, CryLaS GmbH) with a pulse width of 1 ns and a repetition rate of 100 Hz was used. For lifetime measurements by time-correlated single-photon counting, the lasers operated at 200 kHz. The temperature-dependent measurements were performed with a closed-cycle helium cryostat.

### Calculation of the photocarrier density

The corresponding photocarrier density can be calculated as

*ρ*_exc_=light fluence density of a single pulse/(photon energy × optical penetration depth)=4 nJ cm^−2^/(3.07 eV × 1.6 × 10^−19^ J × 220 nm)∼3.7 × 10^14^ cm^−3^

Here the optical penetration depth of CH_3_NH_3_PbBr_3_ is taken as ∼220 nm (ref. 55). In this calculation, we assume a constant excitation because the effective excited volume is remarkably larger than the directly excited one due to the carrier diffusion during the long carrier lifetime^21^. For CH_3_NH_3_PbI_3_ and CH_3_NH_3_PbI_3−*x*_Cl_*x*_, the photon energy of the excitation laser was 1.94 eV, and the optical penetration depth was 250 nm (ref. 1); hence, the photocarrier density was ∼5.2 × 10^14^ cm^−3^.

## Additional information

**How to cite this article:** He, H. *et al.* Exciton localization in solution-processed organolead trihalide perovskites. *Nat. Commun.* 7:10896 doi: 10.1038/ncomms10896 (2016).

## Supplementary Material

Supplementary Information Supplementary Figures 1-11, Supplementary Notes 1-2, Supplementary Methods and Supplementary References.
