# Supplementary material for: Exciton localization in solution-processed organolead trihalide perovskites
Source: Nat Commun. 2016 Mar 21;7:10896. doi: 10.1038/ncomms10896 (PMC4802114; doi:10.1038/ncomms10896)
Supplement: Supplementary Information — Supplementary Figures 1-11, Supplementary Notes 1-2, Supplementary Methods and Supplementary References. [file ncomms10896-s1.pdf]

Supplementary figures

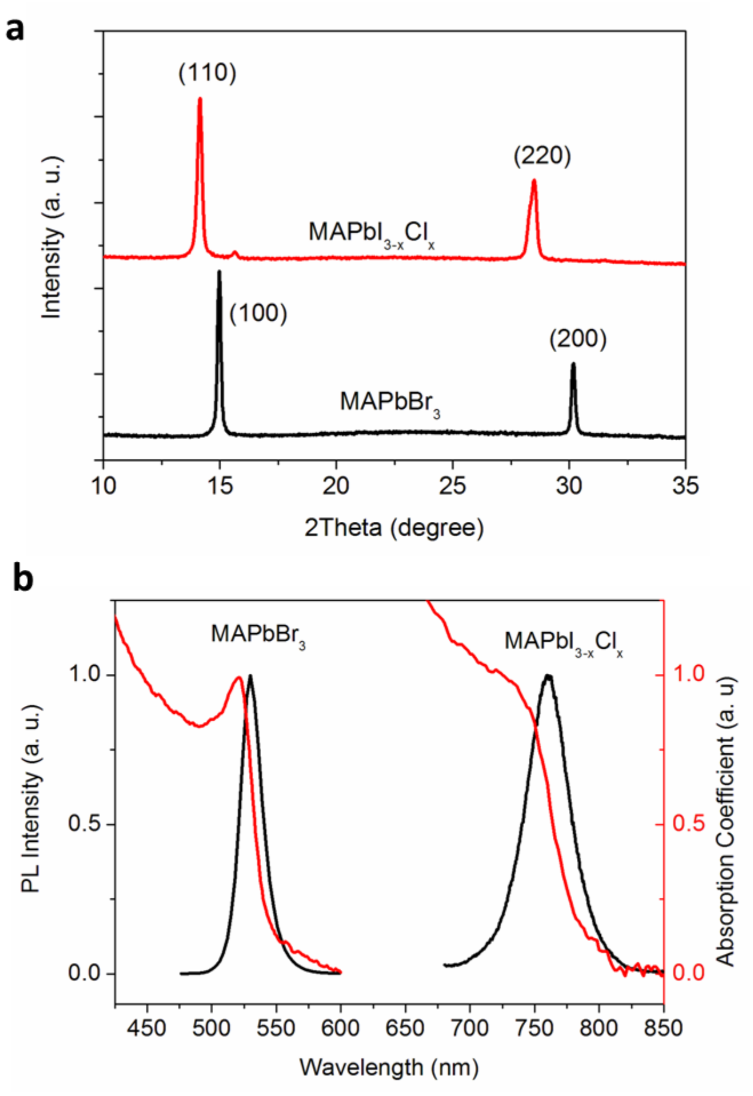

Supplementary Figure 1 | General characterization of solution-processed $CH_3NH_3PbX_3$ films. (**a**) XRD patterns of $CH_3NH_3PbBr_3$ and $CH_3NH_3PbI_{3-x}Cl_x$ films. The weak peak close to 16° is ascribed to cubic $CH_3NH_3PbCl_3$, due to the low chloride solubility limit in the iodide derivative.[1] (**b**) Room temperature optical absorption and photoluminescence spectra of $CH_3NH_3PbBr_3$ and $CH_3NH_3PbI_{3-x}Cl_x$ films. The exciton absorption is clearly seen in $CH_3NH_3PbBr_3$. Both samples show small Stokes shift.

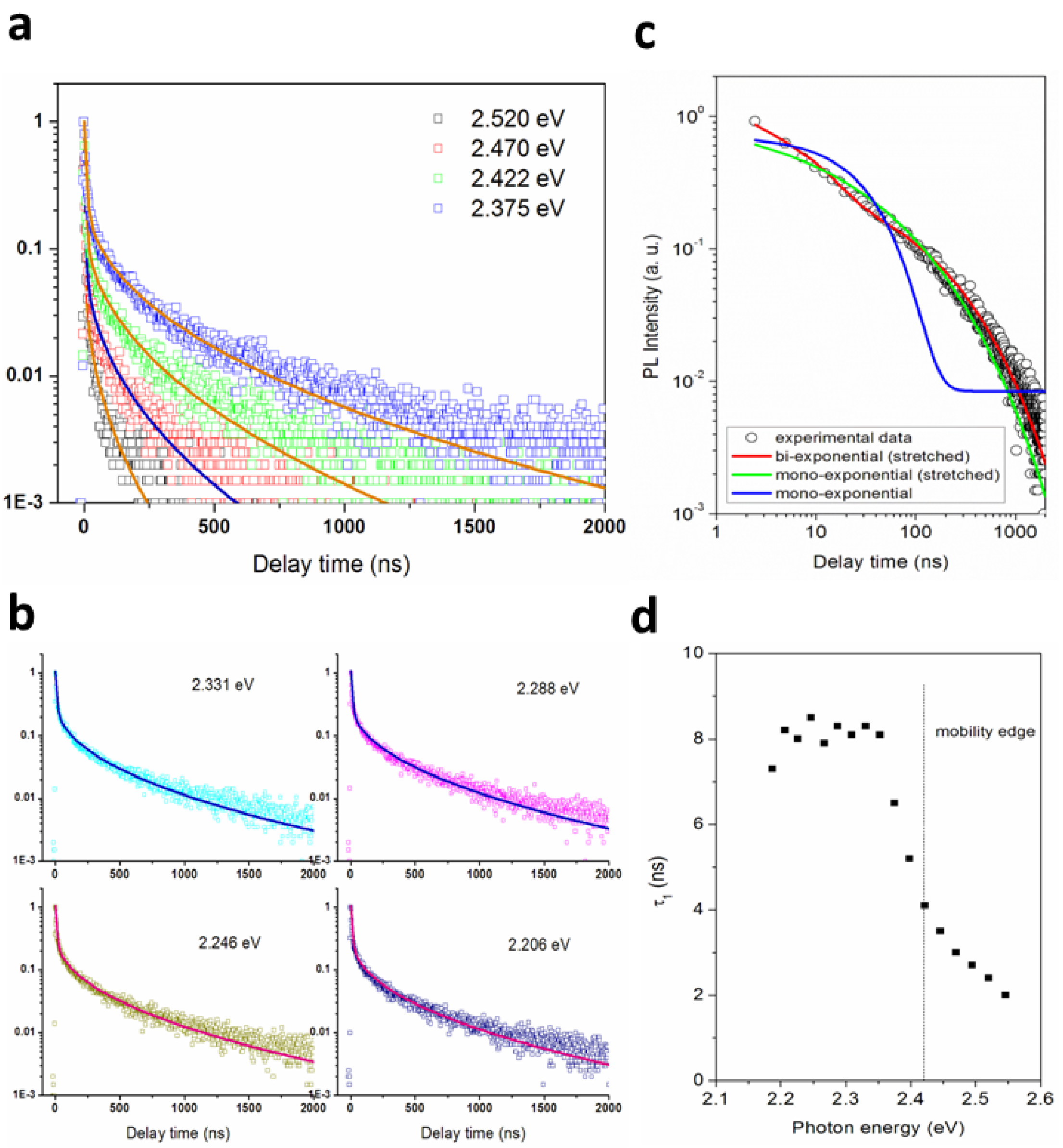

Supplementary Figure 2 │ Fitting results of PL decay of $CH_3NH_3PbBr_3$ films. (**a,b**) Fitting results of decay curves in Fig. 2a using equation (2). (**c**) Fitting results of various exponential lineshapes. The best fit is obtained using bi-exponential (stretched) one (namely, equation (2)). (**d**) The lifetime of localized excitons τ_1 as a function of emission energy, showing features similar to τ_2. τ_1 represents the superposition of both relaxation and recombination of free excitons.

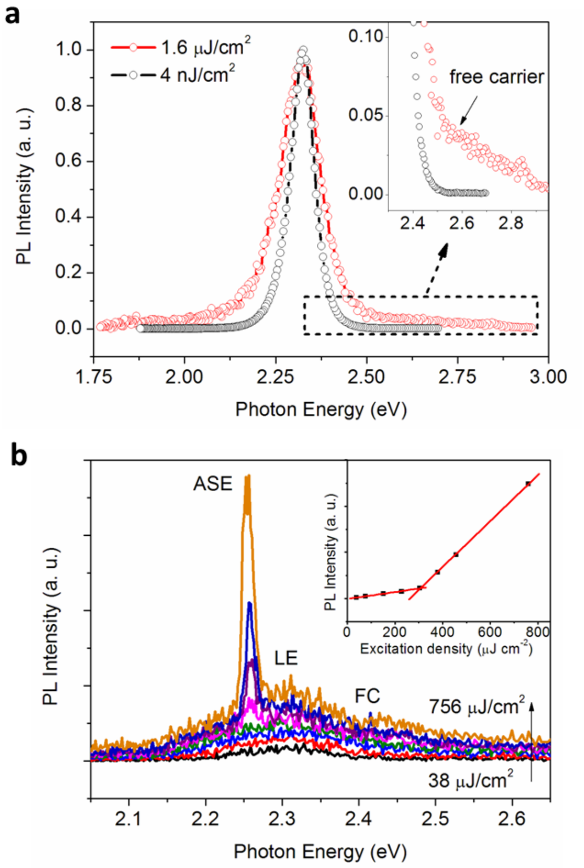

Supplementary Figure 3 │PL spectra of solution-processed $CH_3NH_3PbBr_3$ film under low and high excitation. (**a**) The photocarrier density for the low (black) and moderate (red) excitation is 3.3×10^{14} cm^{-3} and 1.1×10^{17} cm^{-3}, respectively. Inset is the magnified view of the data in dashed rectangle. A high-energy tail due to free carrier recombination is clearly observed under moderate excitation. The peak energy of localized exciton emission is almost unchanged, while the PL broadens due to the dephasing of excitons.[2] (**b**) PL spectra with increasing pump fluence, showing the amplified spontaneous emission (ASE) on the shoulder of localized exciton (LE) emission. Inset plots the integrated intensity of the sharp peak as a function of excitation density, showing the lasing feature. The free carrier (FC) emission around 2.41 eV becomes more and more prominent with increasing pump fluence.

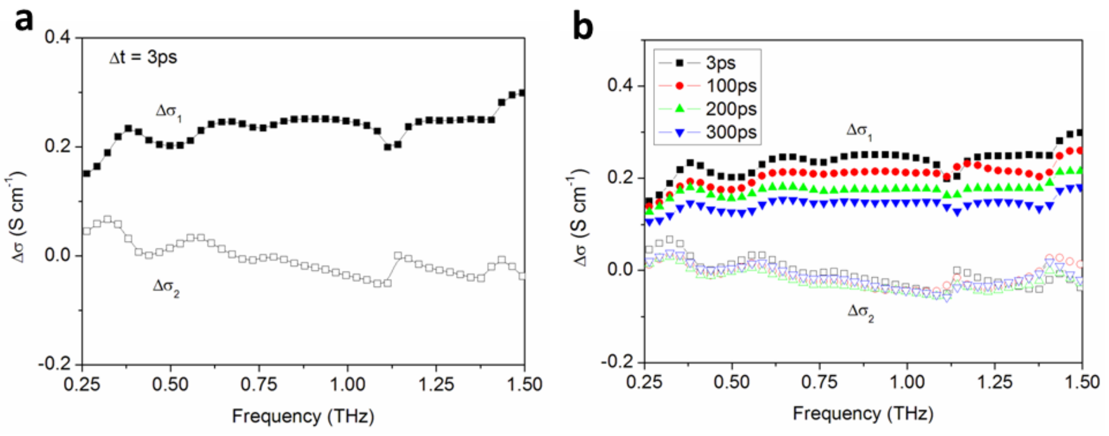

Supplementary Figure 4 | Frequency-dependent THz photoconductivity spectra of $CH_3NH_3PbBr_3$ film. (**a**) The real ($\Delta\sigma_1$) and imaginary part ($\Delta\sigma_2$) of induced photoconductivity immediately after 400 nm laser pump pulses (100 fs, 1kHz repeating frequency). The complex photoconductivity is extracted from the transmitted THz electric field and photo-induced change in THz electric field. The data show a slight but distinguishable decrease of $\Delta\sigma_1$ with decreasing frequency, and negative $\Delta\sigma_2$ at low frequency. The Drude quality factor[3] is calculated to be ~0.96, apparently deviating from the ideal value of 1. The results indicate that our data do not support the free carrier scenario described by the Drude model. In the Drude model for free carriers, the complex photoconductivity is expressed as $\Delta\sigma(\omega) = \frac{\varepsilon_0 \omega_p^2}{\Gamma - i\omega}$, where ω_p is the Drude plasma frequency related to the free carrier density, Γ is the scattering rate of free carriers. The real and imaginary part of photoconductivity can then be written as $\Delta\sigma_1(\omega) = \frac{\varepsilon_0 \omega_p^2 \Gamma}{\Gamma^2 + \omega^2}$ and $\Delta\sigma_2(\omega) = \frac{\varepsilon_0 \omega_p^2 \omega}{\Gamma^2 + \omega^2}$, respectively. Therefore, the Drude model predicts that 1) $\Delta\sigma_1(\omega)$ increases with decreasing frequency and reaches a maximum at zero frequency, and 2) the imaginary part $\Delta\sigma_2(\omega)$ is always positive. The real part increasing with frequency and the negative imaginary part are typical signatures of carrier localization. (**b**) Transient spectra at different delay times. The signatures of carrier localization are maintained.

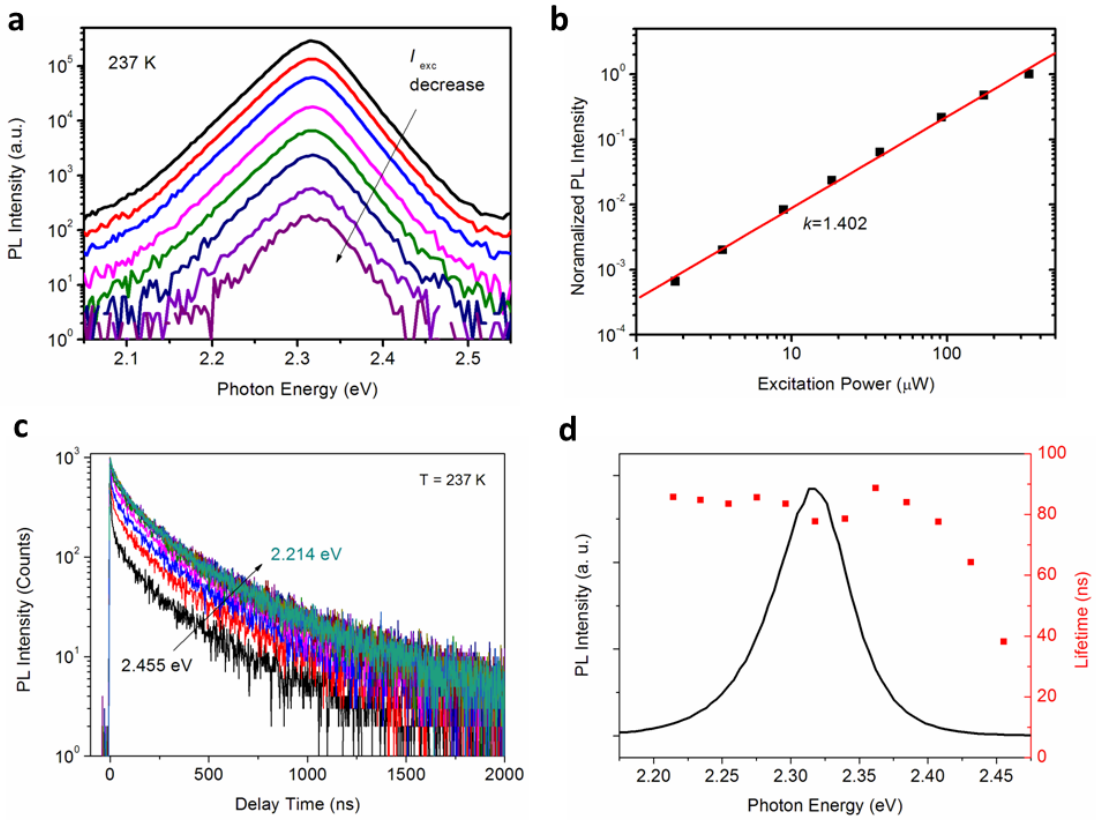

Supplementary Figure 5 │ Evidence for exciton localization in $CH_3NH_3PbBr_3$ film at low temperature. (**a**) Excitation density-dependent PL spectra at 237 K. (**b**) Logarithm plot of integrated PL intensity *vs* excitation density. The data show good power-law dependence with *k* value of 1.402. (**c**) PL decay curves monitored at various emission energy. (**d**) PL lifetime (solid squares) as a function of emission energy. The data show similar trends to that of room temperature illustrated in Fig. 2c.

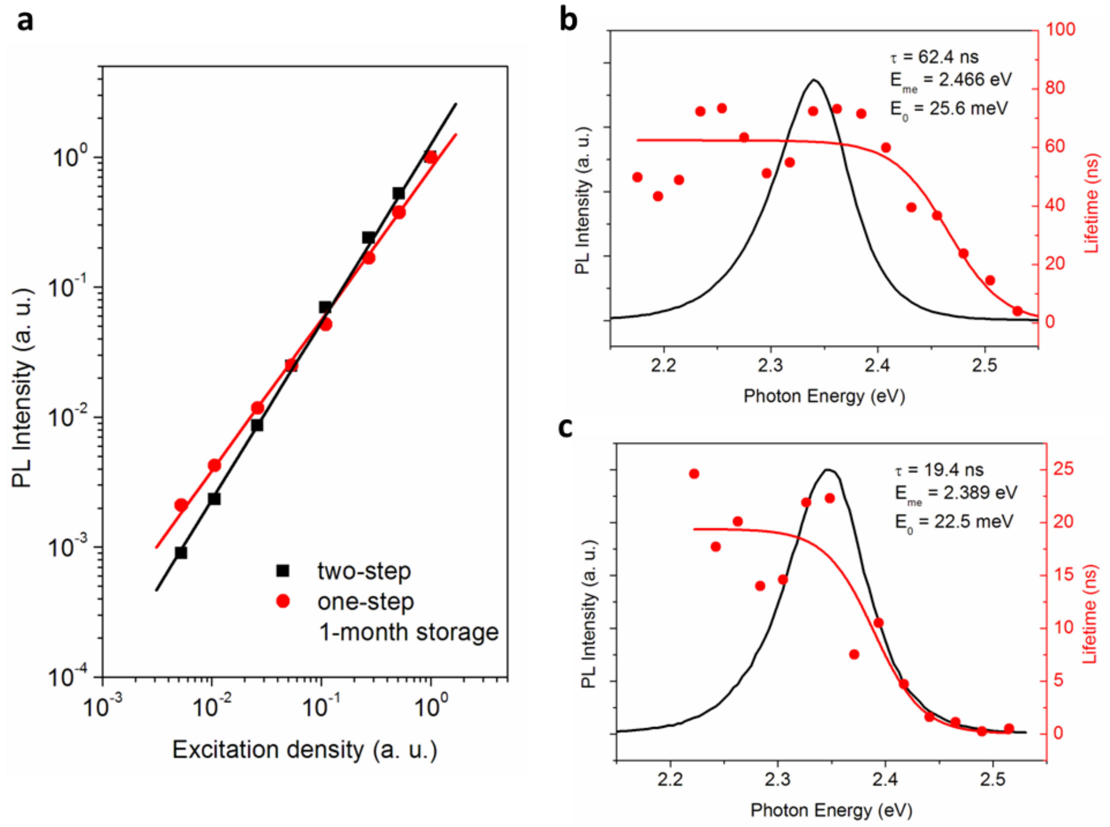

Supplementary Figure 6 | Evidence for exciton localization in different $CH_3NH_3PbBr_3$ films. (**a**) Excitation density-dependent PL intensity of $CH_3NH_3PbBr_3$ films prepared by two-step method[4] and one-step method after 1-month storage. The data show good linearity with *k* value between 1.162 and 1.368. (**b**) Spectral-dependent PL lifetime of $CH_3NH_3PbBr_3$ film prepared by two-step method. (**c**) Spectral-dependent PL lifetime of $CH_3NH_3PbBr_3$ film prepared by one-step method after 1-month storage. The samples show similar features of exciton localization, and the data are fitted with equation (3).

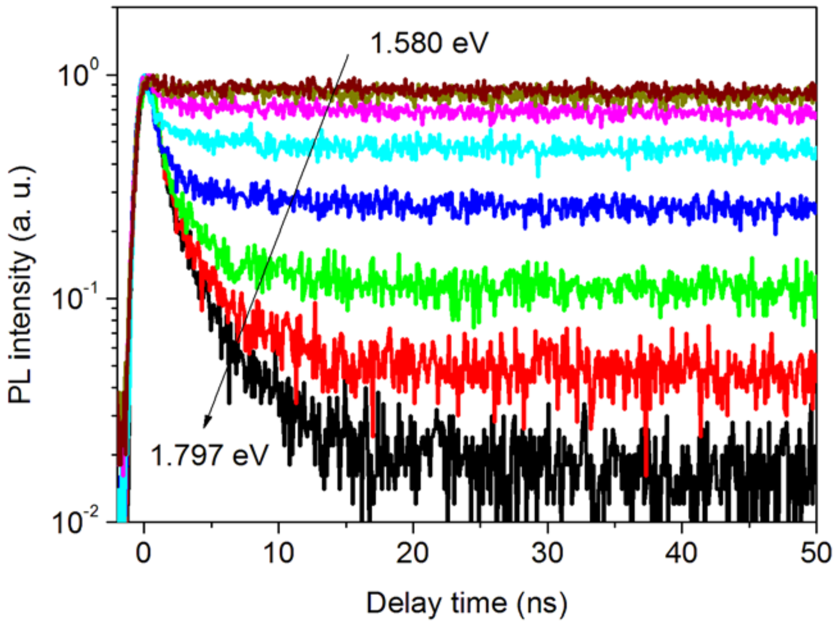

Supplementary Figure 7 │Spectral-dependent PL decay spectra of $CH_3NH_3PbI_{3-x}Cl_x$ film. The decay spectra clearly show fast and slow components. On the high-energy side of PL peak, the lifetime decreases markedly with increasing emission energy.

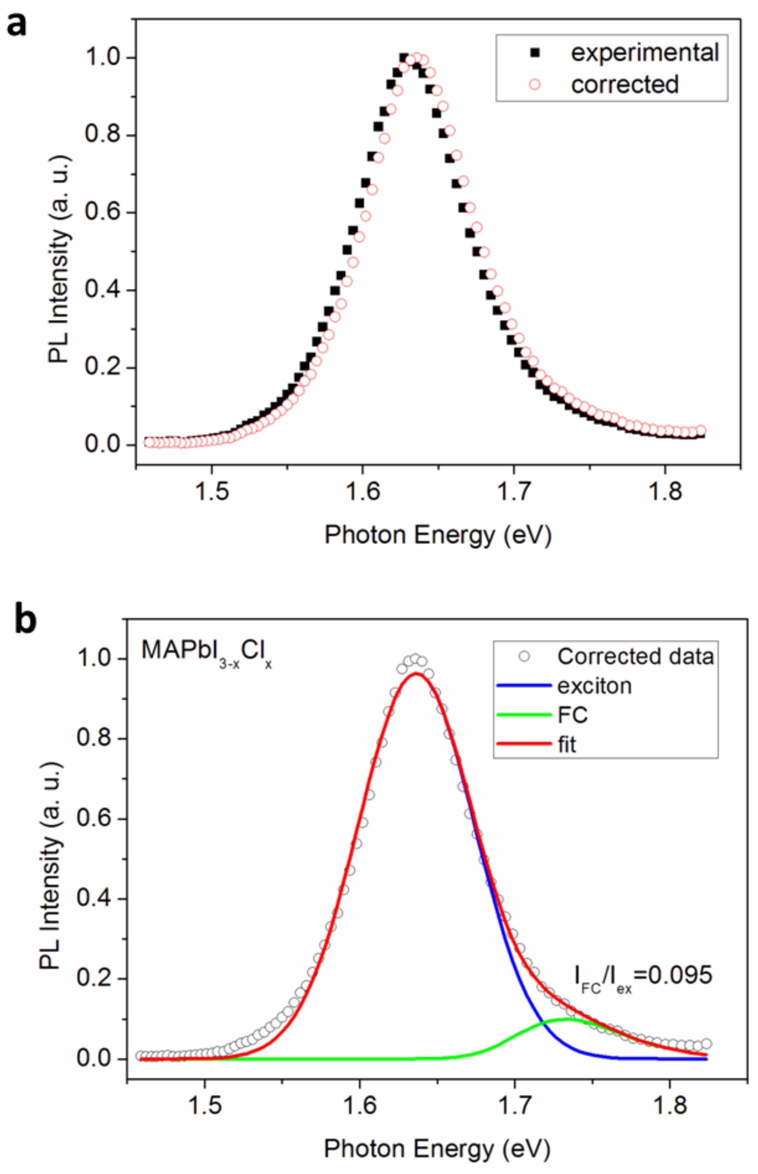

Supplementary Figure 8 │ Lineshape analysis of $CH_3NH_3PbI_{3-x}Cl_x$ PL at room temperature. (**a**) Self-absorption correction of the experimental PL data. The corrected spectrum shows very similar lineshape with a slight blueshift (~5 meV) with respect to the experimental one. (**b**) The spectrum is fitted with exciton emission (Gaussian, blue) plus free carrier (FC) emission (green). The ratio of FC to exciton is calculated as ~0.095. The spectral deviation on the low energy side is due to trapping of excitons. The details of the correction and fitting are described in Supplementary Note 2.

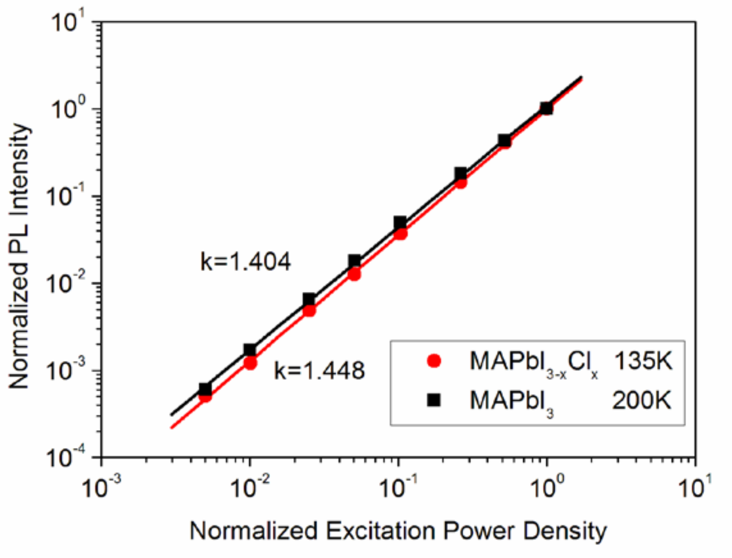

Supplementary Figure 9 | Excitation density-dependent PL intensity of $CH_3NH_3PbI_3$ and $CH_3NH_3PbI_{3-x}Cl_x$ films at low temperature. The data show good power-law dependence with *k* value of 1.404 and 1.448. For both films, the slope is smaller than the room temperature value, 1.569 and 1.513 in Fig. 4a. The excitation density is normalized to the highest value.

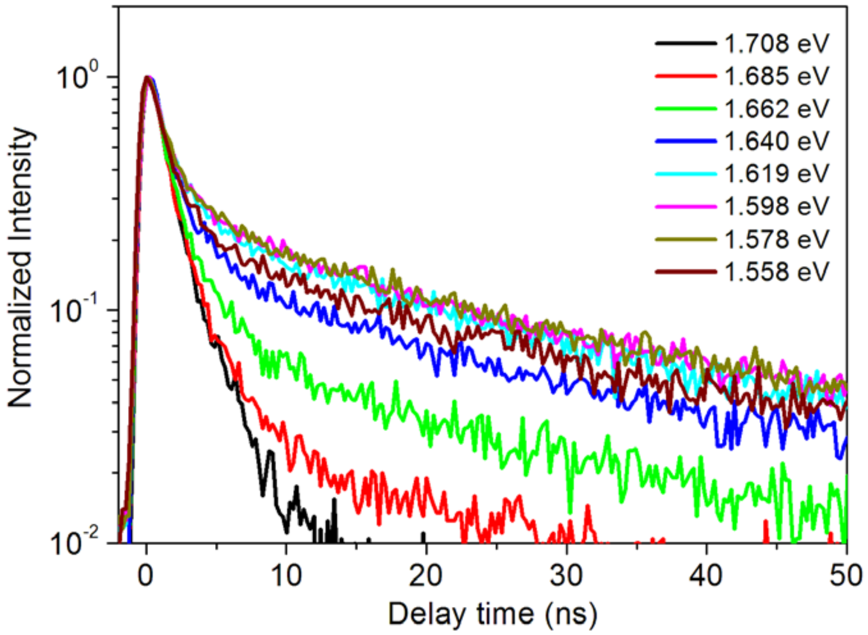

Supplementary Figure 10 │ Spectral-dependent PL decay spectra of photovoltaic device structure based on $CH_3NH_3PbI_{3-x}Cl_x$ film. The photovoltaic device structure is ITO/PEDOT:PSS/Perovskite/PCBM. The decay spectra clearly show fast and slow components. On the high-energy side of PL peak, the lifetime decreases markedly with increasing emission energy.

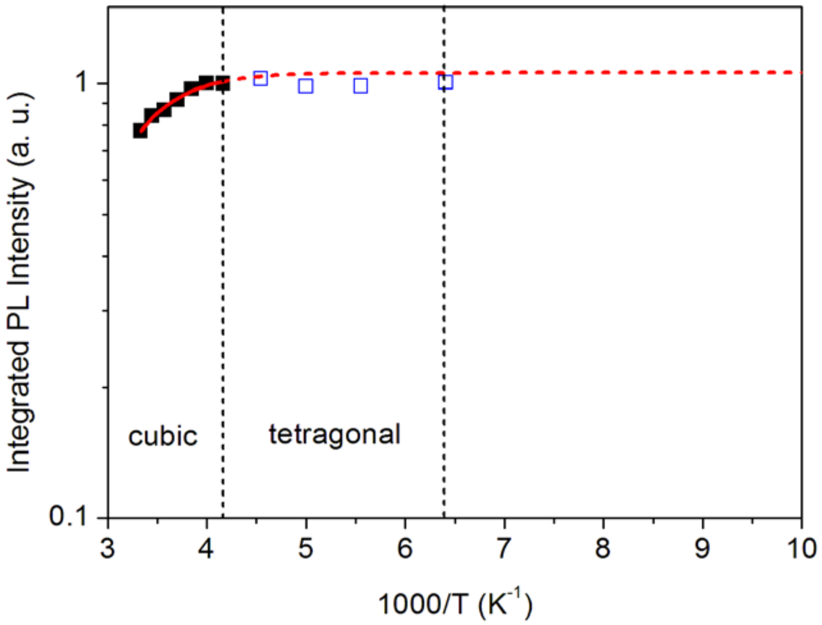

Supplementary Figure 11 │PL thermal quenching behavior of solution-processed $CH_3NH_3PbBr_3$ film under moderate excitation. Integrated PL intensity as function of reciprocal temperature under moderate excitation (photocarrier density ~1×10^{17} cm^{-3}). The data analysis is the same as in Fig. 3.

Supplementary Notes

Supplementary Note 1 Analysis of temperature-dependent PL intensity. The temperature dependence of PL intensity, also known as the thermal quenching of PL, is usually described by[5]

$$I = I_0/[1 + aT^{3/2}\exp(-E_a/kT)] \quad (1)$$

where E_a stands for the thermal activation energy, and k is the Boltzmann constant. It is noteworthy that equation (1) without the item $T^{3/2}$ is also frequently used in the literature. However, such simplified form does not take into account the temperature dependence of the radiative lifetime.[6]

In equation (1), I_0 is the PL intensity at low temperature limit (0 K). In the case of our $CH_3NH_3PbBr_3$ films, however, the low-temperature data cannot be used directly due to the phase transition at 236, 155 and 145 K. By considering the cubic to tetragonal phase transition at 236 K, we first fit the data for cubic phase in Fig. 3 to equation (1). The fitting result is then extrapolated to temperatures lower than 236 K. One can find from Fig. 3 that the expected PL intensity at temperature lower than ~200 K is constant. This assumption is reasonable and supported by the data for the tetragonal phase.

The internal quantum efficiency of PL at certain temperature can then be estimated as IQE (T) = I_{PL}(T)/I_{PL} (0 K). Here the PL intensity at 200 K can be taken as the I_{PL} (0 K). In Fig. 3, the estimated relative IQE at 237 K is ~80 %.

Supplementary Note 2 Lineshape analysis of PL containing free carrier recombination. The PL spectra are firstly corrected for self-absorption using[7]

$$I(E) = \frac{I_{exp}(E)\alpha d}{1-e^{-\alpha d}}, \quad (2)$$

where α is the absorption coefficient, d is the sample thickness. The corrected spectrum is then fitted as the sum of exciton recombination and free carrier (FC) recombination. The lineshape of band-to-band or FC recombination in semiconductors is known as[8,9]

$$I_{FC}(E) \sim (E - E_g)^{1/2} \exp(-(E - E_g)/k_B T) \quad (3)$$

where $I_{FC}(E)$ is the PL intensity, E_g is the band gap, T is the carrier temperature. Aside from direct transitions described by the joint density of states in the bands, there will be Gaussian broadening from phonon interactions that allow indirect transitions. For exciton recombination at RT, the PL lineshape can be well approximated by Gauss function due to the large inhomogeneous broadening. Therefore, equation (3) should be convoluted with a Gaussian to account for such broadening. The entire lineshape is then given by

$$I(E) = I_{ex}(E) + I_{FC}(E) \otimes G(\Gamma), \quad (4)$$

where $I_{ex}(E)$ is a Gaussian to describe the exciton recombination, and $G(\Gamma)$ is a Gaussian of width Γ to account for the broadening from phonon interactions. The best fit is shown in Supplementary Fig. 8b. The intensity ratio of FC to exciton recombination is determined as ~0.095, indicating a small contribution of FC to the overall PL spectra.

Supplementary Methods

Frequency-dependent THz measurements. The sample is characterized by a traditional terahertz time-domain spectroscopy system (THz-TDS). A 100 fs laser beam with a central wavelength of 800 nm, average power of 900 mW and repetition ration of 1 kHz is divided into three portions which are the pump and probe beams for generating and detecting the THz radiation and the control beam for exciting the sample, respectively. The pump beam with 580 mW average power impinges on a <110> ZnTe crystal with 2 mm thickness to launch the linearly polarized THz wave due to the optical rectification. The THz wave is focused on the sample by a parabolic mirror with a focal length of 15 mm and the diameter of the THz focal spot is about 2.5 mm. The transmitted THz wave is collimated and focused on a detection crystal (another <110> ZnTe crystal with 1 mm thickness). The probe beam with 20 mW is

aligned collinearly with the THz wave to illuminates the detection crystal by using a high resistance silicon wafer. Utilizing the electro-optic sampling method,[10] the temporal complex field of the THz wave is obtained. A mechanical chopper and a lock-in amplifier are used to enhance the signal-to-noise ratio of the system. The amplitude and phase of each THz spectrum component is extracted by using the Fourier transformation. The control beam with 300 mW strikes a BaB_2O_4 (BBO) crystal to generate a 400 nm beam with a diameter of 6 mm and an average power of 13 mW. The 400 nm beam illuminates the sample with an incident angle of 30° with respect to the sample surface. The relative time delay between the control beam and THz beam can be exactly controlled with an optical delay line. When the mechanical chopper is inserted into the pump beam, the transmitted THz signals $\tilde{E}_s(\omega)$ and $\tilde{E}_r(\omega)$ from the sample ($CH_3NH_3PbBr_3$ film on a quartz substrate) and its reference substrate (quartz) are obtained. When the chopper is inserted into the control beam, the induced change in the THz signal $\Delta\tilde{E}_s(\omega)$ is measured. Thus, the complex spectrum of the sample under the control beam illumination is $\tilde{E}_{s-pump}(\omega) = \tilde{E}_s(\omega) - \Delta\tilde{E}_s(\omega)$.

Photoconductivity calculation. The complex THz conductivity of the $CH_3NH_3PbBr_3$ film $\tilde{\sigma} = \tilde{\sigma}_1 + i\tilde{\sigma}_2$ can be obtained by the relationships $\tilde{\varepsilon} = 1 + \frac{i}{\omega\varepsilon_0}\tilde{\sigma}$ and $\tilde{\varepsilon} = \tilde{n}^2$, where $\tilde{n}$ is the complex refractive index of the $CH_3NH_3PbBr_3$ film, which can be calculated via the following formula[11]

$$\tilde{T}(\omega) = \frac{2\tilde{n}(\tilde{n}_{sub}+1)\exp[i\omega d(\tilde{n}-1)/c]\exp[-i\omega\Delta L(\tilde{n}_{sub}-1)/c]}{(1+\tilde{n})(\tilde{n}+\tilde{n}_{sub}) + (\tilde{n}-1)(\tilde{n}_{sub}-\tilde{n})\exp(2i\omega d\tilde{n}/c)}, \qquad (5)$$

where $\tilde{T}(w)$ is the complex transmittance of the $CH_3NH_3PbBr_3$ film. The refractive index of the quartz substrate in the frequency range of 0.2-1.5 THz is $\tilde{n}_{sub} = 1.95$, which is measured in the experiment. d and ΔL are the thickness of the $CH_3NH_3PbBr_3$ film and the thickness difference between the sample and reference

substrate. For a very thin metallic film on an insulating substrate, equation (5) can be simplified into a commonly used expression[12]

$$\tilde{T}(\omega) = \frac{(\tilde{n}_{sub} + 1)}{1 + \tilde{n}_{sub} + Z_0 \sigma(\omega) d}, \quad (6)$$

where Z_0 is the free space impedance.

In this experiment, the THz transmittance of the $CH_3NH_3PbBr_3$ film under the control beam illumination is given by $\tilde{T}_{s-pump}(\omega) = \tilde{E}_{s-pump}(\omega) / \tilde{E}_r(\omega)$. The thickness of the samples for THz measurements is ~2.9 μm as determined by cross-sectional scanning electron microscopy (SEM).

The change of the conductivity induced by the control beam $\Delta\tilde{\sigma} = \Delta\tilde{\sigma}_1 + \Delta\tilde{\sigma}_2$ can be obtained by $\Delta\tilde{\sigma}(\omega,\tau) = \tilde{\sigma}_{s-pump}(\omega,\tau) - \tilde{\sigma}_s(\omega,\tau)$.

Supplementary references

1. Colella, S. et al, $MAPbI_{3-x}Cl_x$ mixed halide perovskite for hybrid solar cells: the role of chloride as dopant on the transport and structural properties. *Chem. Mater*. **25**, 4613 (2013).

2. Matsuda, K., Inoue, T., Murakami, Y., Maruyama, S. and Kanemitsu, Y. Exciton dephasing and multiexciton recombinations in a single carbon nanotube. *Phys. Rev. B* **77**, 033406 (2008).

3. Milot, R. L., Eperon, G. E., Snaith, H. J., Johnston, M. B. & Herz, L. M. Temperature-dependent charge-carrier dynamics in $CH_3NH_3PbI_3$ perovskite thin films. *Adv. Funct. Mater.* 25, 6218-6227 (2015).

4. Burschka, J. *et al*. Sequential deposition as a route to high-performance perovskite-sensitized solar cells. *Nature* **499**, 316-319 (2013).

5. Krustok, J., Collan H. & Hjelt K. Does the Low-temperature arrhenius plot of the photoluminescence intensity in CdTe point towards an erroneous activation energy? *J. Appl. Phys.* 81, 1442-1445 (1997).

6. Leroux, M. *et al*. Temperature quenching of photoluminescence intensities in

undoped and doped GaN. *J. Appl. Phys.* **86**, 3721-3728 (1999).

7. Haynes, J. R. New radiation from recombination of holes and electrons in germanium. *Phys. Rev*. **98**, 1866-1868 (1955).

8. Amo, A., Martín, M. D., Viña, L., Toropov, A. I. & Zhuravlev, K. S. Interplay of exciton and electron-hole plasma recombination on the photoluminescence dynamics in bulk GaAs. *Phys. Rev. B* **73**, 035205 (2006).

9. Wang, H., Wong K. S. & Wong G. K. L. Exciton/Free-Carrier Radiative Emission Ratio and Temperature Dependence of Exciton Lifetime for CdZnSSe/ZnSSe Single Quantum Wells. *SPIE* **3624**, 13-24 (1999).

10. Wu, Q., Litz, M. & Zhang, X. C. Broadband detection capability of ZnTe electro-optic field detectors. *Appl. Phys. Lett.* **68,** 2924-2926 (1996).

11. Duvillaret, L., Garet, F. & Coutaz, J. L. Influence of noise on the characterization of materials by terahertz time-domain spectroscopy. *J. Opt. Soc. Am. B* **17,** 452-461 (2000).

12. Averitt, R. D. & Taylor, A. J. Ultrafast optical and far-infrared quasiparticle dynamics in correlated electron materials. *J. Phys.-Condens. Mat.* **14,** R1357-R1390 (2002).
